# MicroRNA Editing Facilitates Immune Elimination of HCMV Infected Cells

**DOI:** 10.1371/journal.ppat.1003963

**Published:** 2014-02-27

**Authors:** Daphna Nachmani, Albert Zimmermann, Esther Oiknine Djian, Yiska Weisblum, Yoav Livneh, Vu Thuy Khanh Le, Eithan Galun, Vaclav Horejsi, Ofer Isakov, Noam Shomron, Dana G. Wolf, Hartmut Hengel, Ofer Mandelboim

**Affiliations:** 1 The Lautenberg Center for General and Tumor Immunology, The BioMedical Research Institute Israel Canada of the Faculty of Medicine, The Hebrew University Hadassah Medical School, Jerusalem, Israel; 2 Department of Virology, Institute for Medical Microbiology and Hygiene, Albert-Ludwigs-Universitat, Freiburg, Germany; 3 Virology Unit, Hadassah Hospital, The Hebrew University Hadassah Medical School, Jerusalem, Israel; 4 Department of Neurobiology, Institute of Life Sciences, The Hebrew University of Jerusalem, Edmond J. Safra Campus, Givat Ram, Jerusalem, Israel; 5 Institute for Virology of the University Hospital Essen, University Duisburg-Essen, Essen, Germany; 6 Goldyne Savad Institute of Gene Therapy, Hadassah-Hebrew University Medical Center, Jerusalem, Israel; 7 Institute of Molecular Genetics, Academy of Sciences of the Czech Republic, Prague, Czech Republic; 8 Sackler Faculty of Medicine, Tel Aviv University, Tel Aviv, Israel; University of Southern California Keck School of Medicine, United States of America

## Abstract

The human cytomegalovirus (HCMV) is extremely prevalent in the human population. Infection by HCMV is life threatening in immune compromised individuals and in immune competent individuals it can cause severe birth defects, developmental retardation and is even associated with tumor development. While numerous mechanisms were developed by HCMV to interfere with immune cell activity, much less is known about cellular mechanisms that operate in response to HCMV infection. Here we demonstrate that in response to HCMV infection, the expression of the short form of the RNA editing enzyme ADAR1 (ADAR1-p110) is induced. We identified the specific promoter region responsible for this induction and we show that ADAR1-p110 can edit miR-376a. Accordingly, we demonstrate that the levels of the edited-miR-376a (miR-376a(e)) increase during HCMV infection. Importantly, we show that miR-376a(e) downregulates the immune modulating molecule HLA-E and that this consequently renders HCMV infected cells susceptible to elimination by NK cells.

## Author Summary

The human cytomegalovirus (HCMV) infects a high percentage of the human population. HCMV infection is life threatening to immune-compromised individuals and when transmitted to the fetus can cause severe birth defects. Thus, it is crucial to understand the anti-viral strategies that are induced in response to HCMV infection. By using molecular tools, next generation deep sequencing of small RNAs and a novel human decidual organ culture we demonstrate that in response to HCMV infection the cell launches an anti-viral response that via miRNA editing enables the elimination of HCMV infected cells. We find that following HCMV infection a specific ADAR1 isoform, ADAR1-p110, is strongly induced and that in addition editing of miR-376a is also increases. We continue to show that the edited-miR-376a then downregulates the inhibitory molecule HLA-E, and by doing so promotes the elimination of HCMV infected cells by NK cells. Finally we demonstrate that in decidual tissues ADAR1-p110 is induced and that HLA-E levels are reduced in response to HCMV infection.

## Introduction

HCMV is a dsDNA herpesvirus, which is highly prevalent in the human population. It establishes its life long latency by using a diverse panel of sophisticated immune evasion strategies, and specifically by manipulating the expression of Human Leukocyte Antigen (HLA) class I molecules [1]–[4]. One of the fascinating examples with this regard is the viral protein UL40 that encodes a signal peptide similar in its sequence to the signal peptide of HLA class I molecules. The UL40 signal peptide is processed and loaded specifically in the groove of HLA-E and thus induces its surface expression, where it can inhibit NK cells function [5], [6].

Natural Killer (NK) cells are innate immune lymphocytes, which recognize and eliminate hazardous cells such as virally infected, transformed and damaged cells. The activity of NK cells is regulated by a balance of signals generated by activating and inhibitory receptors [7]–[10]. While the activating ligands of NK cells are diverse, most of the NK inhibitory ligands belong to the HLA class I family. Thus, reduction in the normal expression levels of HLA class I proteins will result in the activation of NK cells [11], [12]. Among the HLA class I proteins, the non-classical HLA-E is distinct in that it presents in its groove only a limited variety of peptides, which are primarily derived from the signal peptides of other HLA class I molecules [13], [14]. HLA-E is mainly recognized by the inhibitory CD94/NKG2A heterodimer and by the activating CD94/NKG2C receptors, both are expressed by T cells and NK cells [15]. Interestingly, expansion of NK clones expressing NKG2C is often seen HCMV-seropositive individuals [16], yet the function of these NK clones is unclear.

Several activating NK cell receptors exist, that bind to divers ligands. The NKG2D receptor is one of the potent NK cell activating receptors, recognizing 8 different ligands: UPBP 1–6, MICA and MICB [17], [18]. While the ULBP family has orthologous in mice and in primates, mice do not encode MIC genes and most primates encode only a single MIC gene. We have shown in the past that the expression of MICA and MICB is controlled by cellular and by viral miRNAs [19]–[21]. Interestingly, one of the miRNA that was shown by us to target MICB, miR-376a [19], is known to be edited by ADAR enzymes [22].

RNA editing is a post-transcriptional modification that generates diversity in RNA molecules and in proteins. A-to-I RNA editing is catalyzed by the adenosine deaminase acting on RNA (ADAR) enzymes [23], [24], which bind dsRNA structures (that are not completely defined), and can thus edit protein-coding mRNAs and non-coding RNA molecules such as microRNAs (miRNAs). The editing of miRNAs can result in a change in the specificity of the miRNA, especially if the editing event occurs in the seed sequence [24].

Two active ubiquitously expressed ADAR genes are known, ADAR1 and ADAR2 [24]. The ADAR1 enzyme has two considerably different isoforms; ADAR1-p150, which is induced by type I interferon (IFN) [25] and is located mainly in the cytoplasm and ADAR1-p110, that is thought to be constitutively expressed and is located mainly in the nucleus [26]–[28]. ADAR proteins are physiologically crucial as knock-out of Adar1 is embryonic lethal [29]–[31] and knock-out of Adar2 results in behavioral abnormalities including epileptic seizures [32]. ADAR proteins also function during viral infections and were shown to be either proviral or antiviral [33].

Here we show that specifically during HCMV infection ADAR1-p110, and not ADAR1-p150, is induced. We further show that increased editing of miR-376a is observed upon HCMV infection and that the edited miR-376a (miR-376a(e)), downregulates the expression of HLA-E to render infected cells susceptible to elimination by NK cells.

## Results

### ADAR1-p110 is induced during HCMV infection via a specific promoter

**Figure 1:**
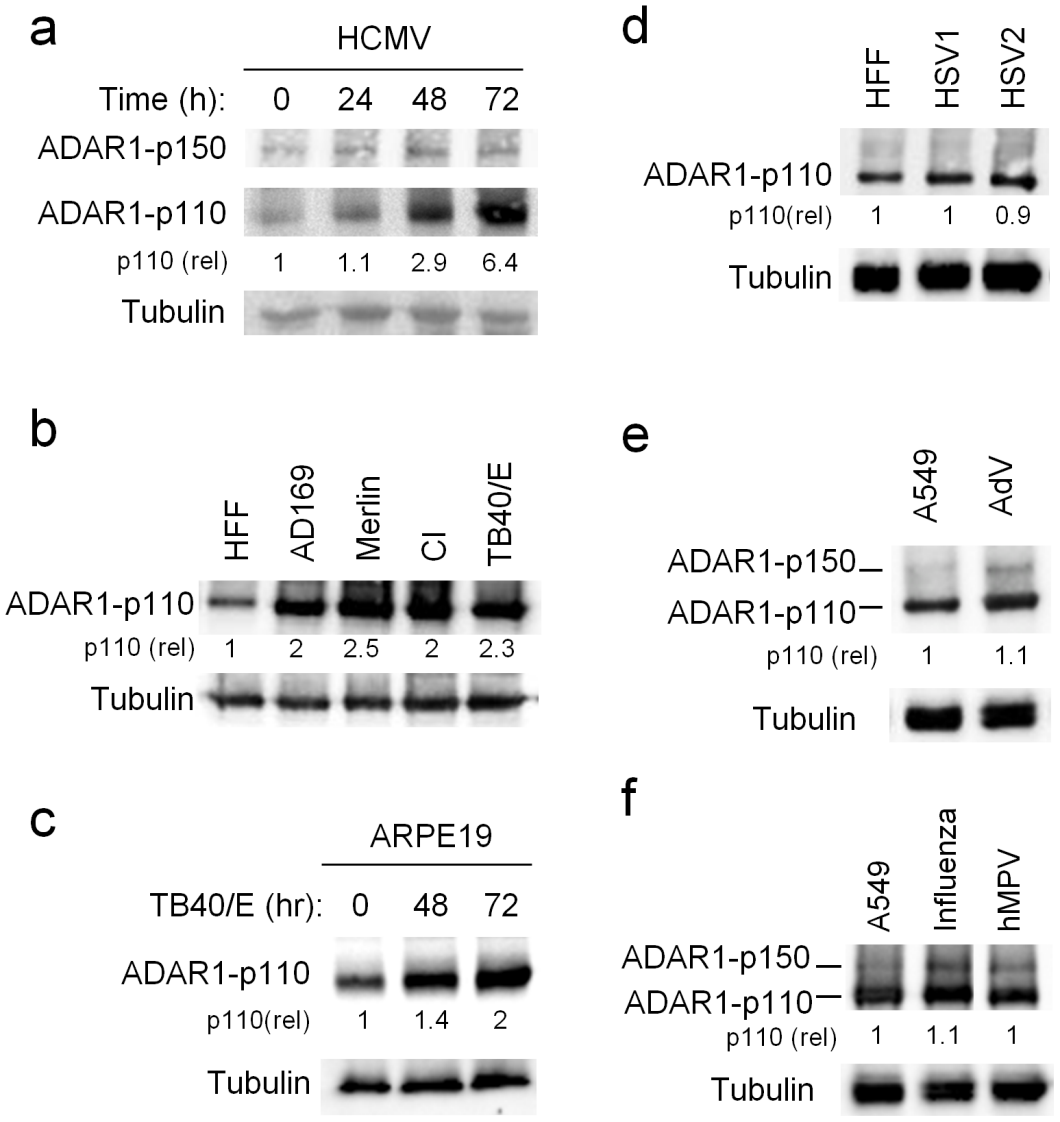
ADAR1-p110 is induced during HCMV infection. (a–f) WB analysis of ADAR1 expression during various viral infections. (a) Time course analysis of ADAR1-p150 and ADAR1-p110 expression during infection of HFF cells with the AD169 strain of HCMV. Quantification of ADAR1-p110 was performed relative to expression at time 0 h (the fold increased numbers are indicated). Data are representative of four independent experiments. (b) Analysis of ADAR1-p110 expression during infection of HFF cells with the indicted HCMV strains. The analysis was performed 48 hrs after infection. Quantification of ADAR1-p110 was performed relative to expression in uninfected HFF cells (the fold increased numbers are indicated below the blot). Data are representative of three independent experiments. CI – clinical isolate (c) Analysis of ADAR1-p110 expression at different time points following infection of ARPE-19 cells with the TB40/E strain of HCMV. Quantification of ADAR1-p110 was performed relative to expression at time 0 h (the fold increased numbers are indicated below the blot). Data are representative of four independent experiments. (d) Analysis of ADAR1 expression during infection of HFF cells with HSV1 and HSV2, 24 hrs after infection. Quantification of ADAR1-p110 was performed relative to expression in uninfected HFF cells (the fold increased numbers are indicated below the blot). Data are representative of three independent experiments. (e and f) Analysis of ADAR1 expression during infection of A549 cells with Adenovirus (AdV, e), Influenza (f) and human metapneumovirus (hMPV, f), 48 hrs and 96 hrs after infection, respectively. Quantification of ADAR1-p110 was performed relative to expression in uninfected A549 cells (the fold increased numbers are indicated below the blot). Data are representative of three independent experiments. (a–f) Alpha-tubulin served as a loading control.

Because it is known that ADAR1-p150 is induced during several viral infections [34]–[37] and since it is unknown whether ADAR proteins are induced during HCMV infection we initially analyzed the expression of the two active ADAR proteins, ADAR1 and ADAR2, prior and following HCMV infection of Human Foreskin Fibroblasts (HFFs). ADAR2 was undetected in HFF cells prior (Fig. S1a), and following infection (data not shown). Surprisingly, although it is known that the expression of ADAR1-p150 is induced following interferon (IFN) treatment [25] (Fig. S1b, quantified in Fig. S1c), only the expression of ADAR1-p110, which was considered until now constitutive, significantly increased following HCMV infection (Fig. 1a, quantified in Fig. S1d).

Next, we investigated whether ADAR1-p110 would be induced by additional HCMV strains. We infected cells with AD169, TB40/E, low passage Merlin strain, and with a clinical isolate (CI) and observed increased expression of ADAR1-p110 following infection by all strains (Fig. 1b, quantified in Fig. S1e). The induction of ADAR1-p110 was not cell specific as it was observed also in ARPE-19 cells (epithelial origin) infected with the TB40/E strain (Fig. 1c, quantified Fig. S1f). Next, to examine whether the induction of ADAR1-p110 is virus specific, we infected HFF cells with additional herpesviruses; HSV1 and HSV2, and did not observe any changes in ADAR1-p110 expression (Fig. 1d, quantified in Fig. S1g). We also infected A549 cells with the DNA virus Adenovirus (AdV, Fig. 1e, quantified in Fig. S1h), and with the RNA viruses Influenza and the human metapneumovirus (hMPV, Fig. 1f, quantified in Fig. S1i). We used A549 in these cases, as HFF cells are not permissive to infections with these viruses. Following infection we did not observe changes in ADAR1-p110 expression (Fig. 1e and f quantified in Fig. S1h and I, respectively). Yet, the expression of ADAR1-p150 was increased by these viruses (Fig. 1e and f quantified in Fig. S1h and I, respectively). Thus we conclude that following HCMV infection ADAR1-p110 is specifically induced.

### ADAR1-p110 is induced via specific promoter during HCMV infection

**Figure 2:**
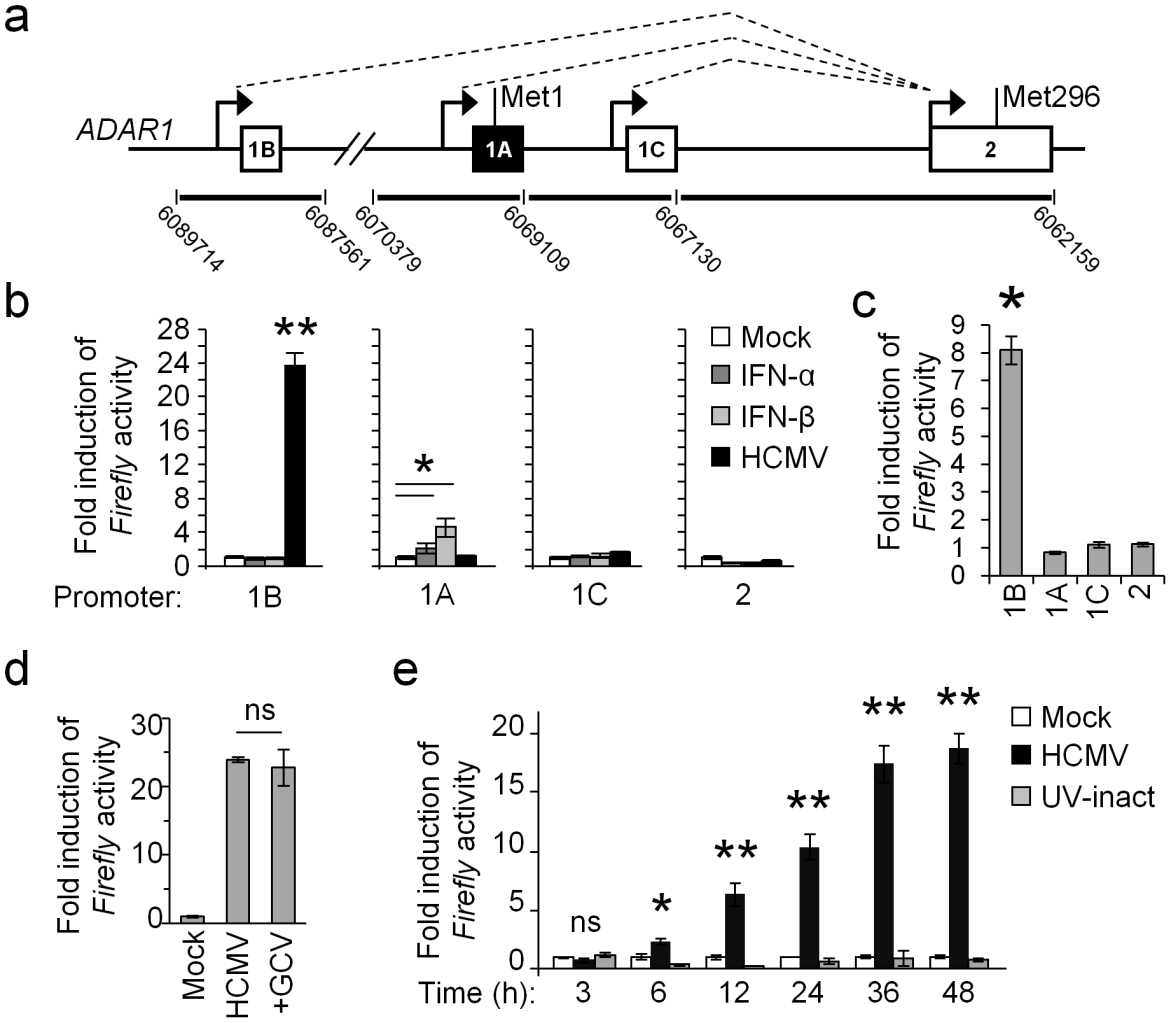
ADAR1-p110 is induced via a specific promoter. (a) Schematic description of the alternative promoters of the ADAR1 gene (black arrows) and their alternative splicing (dotted lines). Exons 1B, 1C and 2 (white boxes), drive the expression of the ADAR1-p110, while exon 1A (black box) drives the expression of ADAR1-p150. The genomic locations of the DNA fragments, which were cloned upstream to a Firefly luciferase, are indicated at the bottom. (b) Dual luciferase assay was performed on HFF cells that were transfected with the reporter vectors containing different genomic regions of the ADAR1 promoters as indicated, 4 hrs after transfection the cells were either mock treated (Mock) or treated with IFN-α (1000 u/ml), IFN-β (1000 u/ml), or infected with HCMV (at MOI 1) for 48 hours. The Firefly/Renilla ratio of each treatment was normalized to the ratio in mock HFF cells. Data are representative of four independent experiments, shown are mean ± S.D. of triplicates. *P<0.01, **P<5E-5 by two-tailed Student's t-test. (c) ARPE-19 cells were transfected with the indicated reporters (x-axis). Four hours after transfection the cells were infected with the TB40/E strain of HCMV. 48 hrs after infection the Firefly/Renilla ratio was assessed and calculated relatively to the activity in the uninfected cells. Data are representative of four independent experiments; shown are mean ± S.D. of triplicates. *P<0.001, by Student's t-test. (d) HFF cells were transfected with the 1B reporter, and then infected with HCMV in the presence or absence of the inhibitor GCV. Firefly/Renilla activity ratio was assessed 48 hrs after infection, and was calculated relatively to the activity in the uninfected calls. Data are representative of three independent experiments; shown are mean ± S.D. of triplicates. ns-not significant. (e) HFF cells were transfected with the 1B reporter. Firefly/Renilla activity ratio was assessed at the indicated time points in mock treated cells (Mock), in cells infected with HCMV, or in cells infected with an UV-inactivated virus (UV-inact). The Firefly/Renilla ratio was calculated relatively to the ratio in mock HFF cells. Data are representative of three independent experiments; shown are mean ± S.D. of triplicates. *P<0.002, **P<0.0003, by Student's t-test.

To elucidate the mechanism of ADAR1-p110 induction we cloned all known *ADAR1* promoters upstream to a Firefly reporter. Expression of the *ADAR1* gene is controlled by four alternative promoters, of which three drive the expression of ADAR1-p110 (Fig. 2a, genomic regions 1B, 1C and 2, white boxes, translation initiation at Met296) and a fourth promoter, IFN-inducible, that controls the expression of ADAR1-p150 (Fig. 2a, genomic region 1A, black box, translation initiation at Met1). The activity of each reporter was assessed under IFN-α or IFN-β treatments or during HCMV infection (Fig. 2b). As previously reported, the genomic region 1A induced the reporter's activity following IFN treatment [25] (Fig. 2b). Importantly, while genomic regions 1C and 2 did not affect the activity of the Firefly reporter in the various treatments (Fig. 2b), genomic region 1B strongly induced Firefly activity, only following HCMV infection (Fig. 2b). We also assessed the activity of the various reporters in ARPE-19 cells infected with TB40/E strain and obtained similar results (Fig. 2c)

To investigate at which stage of infection is ADAR1-p110 induced, we transfected HFF cells with the 1B reporter, and then infected the cells in the presence or absence of late-gene expression inhibitor, Ganciclovir (GCV, Fig. 2d). As can be seen, GCV treatment did not affect ADAR1-p110 induction (Fig. 2d). Indeed when we followed the kinetics of ADAR1-p110 induction we observed that the induction of the 1B reporter's expression was very rapid, and was detected as early as six hours post infection (Fig. 2e, black bars). Moreover, it was not induced when a UV-inactivated virus was used (Fig. 2e, grey bars), indicating that the 1B promoter is activated only due to infection by a transcriptionally active virus, either by the virus itself or as a cellular response to HCMV infection.

### ADAR1-p110 affects the editing of miR-376a

**Figure 3:**
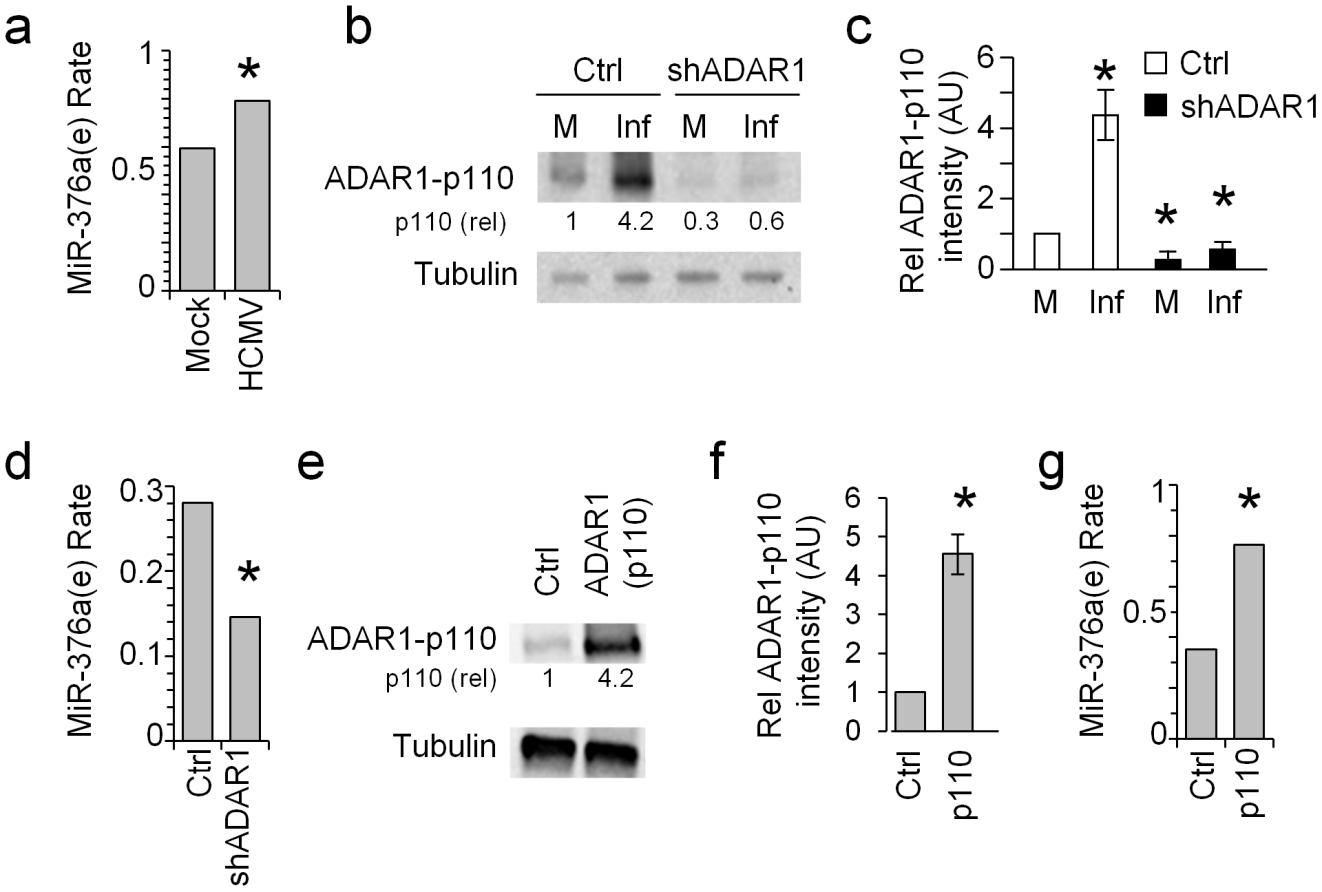
ADAR1-p110 is involved in the editing of miR-376a. (a) The rate of miR-376a(e) in HFF cells either mock treated or HCMV infected was determined by next generation small RNA deep sequencing. Data are combined of two independent experiments. The rate in mock treated cells: 0.5943, in HCMV infected cells: 0.7912, *P-value: 4.38E-13. The rate of miR-376a(e) was calculated as the proportion of miR-376a(e) out of all the miR-376a species after allowing two mismatches. Hence no specific normalization was required. (b and c) WB analysis (b) and quantification (c) of ADAR1-p110 expression in mock treated or HCMV infected HFF cells transduced either with shADAR1 vector or with a control vector. (b) Data are representative of three independent experiments. Numbers indicate fold change in intensity compared to the expression of ADAR1-p110 observed after mock infection (M) in the control vector transduced cells. (c) Quantification of all experiments performed in (b). Shown are the relative average intensities ± S.D., *P<0.001 by Student's t-test. (d) The rate of miR-376a(e) in HFF cells transduced either with a control vector (Ctrl) or shADAR1, and HCMV infected was determined by next generation small RNA deep sequencing. Data are combined of two independent experiments. The rate in control cells: 0.2806, in shADAR1 cells: 0.0.1461, *P-value: 1.6E-4. The rate of miR-376a(e) was calculated as the proportion of miR-376a(e) out of all the miR-376a species after allowing two mismatches. Hence no specific normalization was required. (e and f) WB (e) and quantification (f) of ADAR1-p110 expression in ADAR1-p110-transduced HFF cells. Data are representative of three independent experiments. Numbers indicate fold change in intensity of ADAR1-p110 compared to HFF cells transduced with an empty vector. (f) Shown are the relative average intensities of ADAR1-p110±S.D., *P<0.002 by Student's t-test. (g) The rate of miR-376a(e) in HFF cells transduced either with a control vector or ADAR1-p110 was determined by next generation small RNA deep sequencing. Data are combined of two independent experiments. The rate in control cells: 0.3517, in ADAR1-p110 cells: 0.7653, *P-value: 3.97E-50. The rate of miR-376a(e) was calculated as the proportion of miR-376a(e) out of all the miR-376a species after allowing two mismatches. Hence no specific normalization was required.

MiR-376a was shown to be one of the primary miRNAs that undergo editing by ADAR proteins [22]. Here we observed that ADAR1-p110 is specifically induced during HCMV infection (Figs. 1 and 2). Thus, we were next interested to evaluate the levels of the edited-miR-376a (named here miR-376a(e)) during HCMV infection. As miR-376a and miR-376a(e) differ in only one nucleotide, it is impossible to distinguish between the two miRNAs by qRT-PCR. Hence we performed next generation small RNA sequencing on uninfected and HCMV infected HFF cells and observed an increase in miR-376a(e) levels following infection (Fig. 3a).

To demonstrate that ADAR1-p110 has a role in the editing of miR-376a during HCMV infection we knocked down ADAR1. Because the transcript of ADAR1-p110 is included within the transcript of ADAR1-p150 it is impossible to knock down only ADAR1-p110 without affecting the expression of ADAR1-p150. We therefore prepared two shRNA constructs, one that targets ADAR1-p150 specifically (shADAR1-p150) and another that targets both isoforms of ADAR1 (shADAR1) and transduced HFF cells with these constructs. The KD of ADAR1-p110 by shADAR1 was very efficient as demonstrated by WB analysis of uninfected and HCMV infected HFF cells (Fig. 3b, quantified in Fig. 3c), while the KD of ADAR1-p150 affect only the expression of ADAR1-p150 (Fig. S2). Next, we evaluated the levels of miR-376a(e) in the shADAR1 cells following HCMV infection by small RNA next generation sequencing and observed reduced levels of miR-376a(e) as compared to control cells (Fig. 3d).

To corroborate the above findings, we cloned ADAR1-p110 into a lentiviral vector and transduced HFF cells. The over expression of ADAR1-p110 was validated by WB (Fig. 3e, quantified in Fig. 3f). We analyzed the levels of miR-376a(e) by small RNA next generation sequencing and observed increased levels of miR-376a(e) specifically in ADAR1-p110-expressing HFF cells (Fig. 3g).

### Editing of miR-376a abolishes its ability to regulate MICB

**Figure 4:**
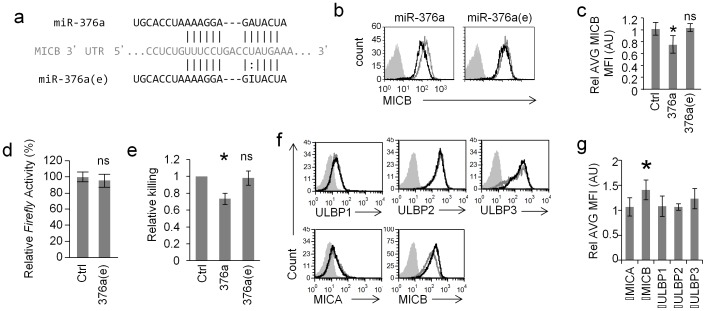
RNA editing of miR-376a abolishes its regulation of MICB. (a) Alignment of miR-376a (black top) and miR-376a(e) (black bottom) to the 3′ UTR of MICB (grey). Solid lines represent Watson-Crick base pairing; dotted lines represent wobble base pairing. (b and c) FACS analysis (b) and quantification (c) of the expression of MICB on RKO cells transduced with miR-376a (black histogram left panel), or with miR-376a(e) (black histogram right panel) or with a control miRNA (grey histogram). The filled grey histogram represents background staining. Data is a representative of four independent experiments. (c) Quantification of all experiments performed in (b). Shown are relative average MFI ± S.D. *P<0.03 by Student's t-test, n.s. not-significant. (d) Dual luciferase assay was performed on RKO cells transduced either with miR-376a(e) or with a control miRNA. The various RKO cells were transfected with a luciferase reporter plasmid that was fused to the 3′ UTR of MICB. Data are representative of five independent experiments. Shown are mean ± S.D. of triplicates. ns-not significant. (e) Killing of RKO cells transduced with the indicated miRNAs, by bulk NK cells. Data are mean ± S.D of three independent experiments (*P<0.01, by Student's t-test). (f) FACS analysis of NKG2D ligands expressed on RKO cells transduced with ADAR1-p110 (black histogram) or a control vector (grey empty histogram). Filled grey histogram is background staining. Data is a representative of four independent experiments. (g) Quantification of all experiments performed in (f). Shown are relative average MFI ± S.D. *P<0.02 by Student's t-test.

So far we showed that ADAR1-p110 is induced specifically during HCMV infection and that ADAR1-p110 can edit miR-376a. Interestingly, we demonstrated in the past that miR-376a down regulates the expression of the stress-induced ligand MICB [19]. Because the editing of miR-376a occurs in its seed region, it is expected (as previously reported [22]) that the target spectrum of miR-376a(e) will be different than that of miR-376a. Indeed, miR-376a(e) does not have a full seed base-pairing with the 3′UTR of MICB (Fig. 4a). Thus, we next tested whether miR-376a(e) is able to control the expression of MICB.

RKO cells were transduced with lentiviral vectors encoding miR-376a, miR-376a(e), or a control miRNA. As previously reported [19], a moderate, yet statistically significant reduction in MICB expression was observed in cells transduced with miR-376a (Fig. 4b, left, quantified in Fig. 4c), while the expression of other NKG2D ligands expressed on RKO cells was not affected (Fig. S3a). In contrast, little or no change in MICB levels was observed in cells transduced with miR-376a(e) (Fig. 4b right, quantified in Fig. 4c). Furthermore, dual luciferase reporter assays demonstrated that miR-376a(e) is unable to repress reporter's activity when fused to the 3′ UTR of MICB (Fig. 4d). These observations were supported by NK killing assays against the transduced RKO cells in which the expression of miR-376a lead to reduced NK cell killing while expression of miR-376a(e) had no effect (Fig. 4e). Thus, we concluded as we previously reported [21], [38] that even a moderate change in MICB expression that is mediated by miR-376a is sufficient to affect NK cytotoxicity and that miR-376a(e) does not affect MICB expression. In light of future findings describe below, we also assessed the levels of HLA-E on RKO cells and found that they do not express HLA-E (Fig. S3b).

We next transduced RKO cells with lentiviral vectors expressing either ADAR1-p110 or a control vector and assessed the levels of the NKG2D ligands (Fig. 4f, quantified Fig. 4g). Although RKO cell already express ADAR1 (Fig. S4a) overexpression of ADAR-p110 (black histograms), moderately elevated the levels of MICB only (Fig. 4f, quantified in Fig. 4g), probably due to reduced miR-376a levels. As an additional control we also overexpressed ADAR1-p110 in cells that do not express or express little amounts of MICB (293T and BJAB, but express ADAR1-p110, Fig. S4a) and observed no changes in the expression of MICB or in other NKG2D ligand (Fig. S4b). Thus, we concluded that editing of miR-376a abolishes its ability to regulate the expression of MICB.

### MiR-376a(e) directly binds the 3′ UTR of HLA-E and regulates its expression

**Figure 5:**
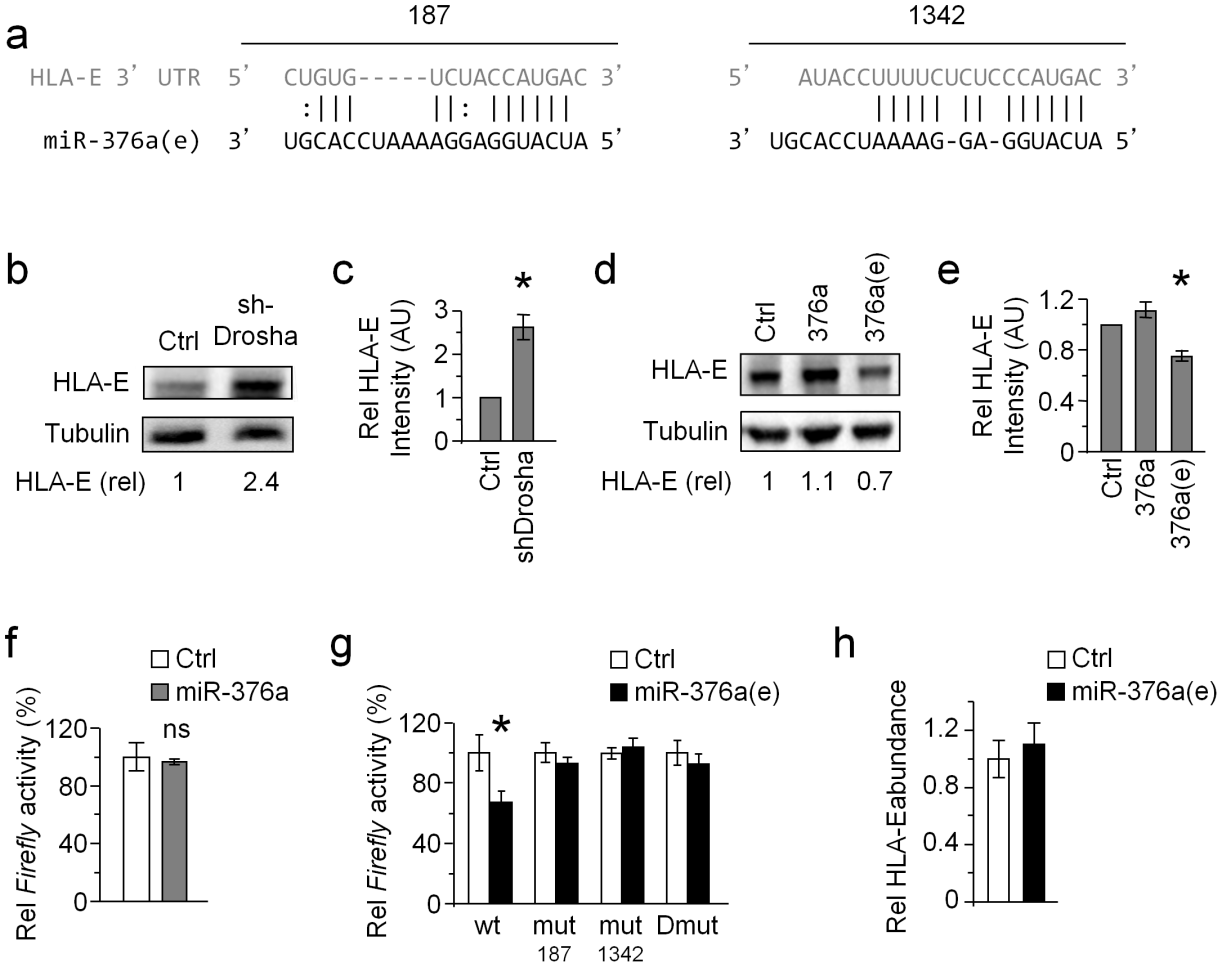
HLA-E is regulated by miR-376a(e). (a) Alignment of the two predicted binding sites of miR-376a(e) (in black) in the 3′ UTR of HLA-E (grey). The locations of the binding sites in the 3′ UTR of HLA-E are indicated above the alignment. (b and c) WB analysis (b) and quantification (c) of HLA-E expression in HeLa cells transduced either with shDrosha or with a control vector (top). Numbers indicate fold induction of HLA-E. Data are representative of three independent experiments. (c) Quantification of all experiments performed (b). Shown are the relative average intensities ± S.D of the shDrosha compared to control that was set as 1. *P<0.008, by Student's t-test. (d and e) WB analysis (d) and quantification (e) of HLA-E expression in BJAB cells transduced with miR-376a(e), miR-376a, or a control miRNA. Numbers indicate fold induction of HLA-E. Data are representative of three independent experiments. (e) Quantification of all experiments performed (d). Shown are relative average intensities ± S.D compared to control that was set as 1. *P<0.001, by Student's t-test. (f) 293T cells expressing either control miRNA or miR-376a were transfected with a luciferase reporter fused to the 3′ UTR of HLA-E. Data are representative of four independent experiments; shown are average mean ± S.D. of triplicates. ns – not significant. (g) The various reporter plasmids (indicated in the x axis), were transfected to 293T cells expressing either miR control or miR-376a(e). Data are representative of five independent experiments; shown are average mean ± S.D. of triplicates. *P<0.01, by Student's t-test. (h) Quantitative real-time PCR analysis of the relative abundance of HLA-E mRNA in 293T cells transduced either with miR-376a(e) or with a control miRNA. GAPDH was used as a reference gene. Data are representative of two independent experiments; shown are average mean ± SEM of triplicates.

Because miR-376a(e) does not control the expression of MICB (Fig. 4) and since we showed that ADAR1-p110 is strongly induced specifically upon HCMV infection (Figs. 1 and 2) leading to increase levels of miR-376a(e) (Fig. 3). We next wondered whether miR-376a(e) might regulate the expression of immune genes that are known to be affected during HCMV infection. To that end we utilized the online algorithm RNAhybrid (http://bibiserv.techfak.uni-bielefeld.de/rnahybrid/submission.html) to screen for possible miR-376a(e) binding sites in 3′UTR of various related genes. One of the top candidates was HLA-E, which was predicted to have two binding sites for miR-376a(e) in its 3′ UTR (Fig. 5a), yet no such sites were predicted for the unedited miR-376a.

Whether HLA-E is regulated by miRNAs is unknown. Therefore, we initially tested this by knocking down the RNase III enzyme Drosha (one of the central enzymes involved miRNA biogenesis [39]). KD of Drosha (which was validated by WB, see Fig. S5a, quantified in Fig. S5b) resulted in elevated levels of HLA-E (Fig. 5b, quantified in Fig. 5c).

To demonstrate that miR-376a(e) specifically, controls the expression of HLA-E we overexpressed miR-376a(e), miR-376a or a control miRNA in BJAB cells (we selected BJAB cells because they express HLA-E). Indeed reduced HLA-E levels were observed only in cells transduced with miR-376a(e), whereas the unedited miR-376a or control-miRNA had no effect (Fig. 5d, quantified in Fig. 5e).

To validate that miR-376a(e) regulates HLA-E by directly binding to the predicted binding sites, we fused the 3′ UTR of HLA-E downstream to a *Firefly* reporter. Dual luciferase assays were performed in cells transduced with lentiviruses expressing miR-376a(e), miR-376a or control miRNA and then transfected with the *Firefly* reporter. While expression of miR-376a did not affect the reporter's activity (Fig. 5f), repression was observed in cells expressing miR-376a(e) (Fig. 5g). To demonstrated that miR-376a(e) regulates HLA-E expression by direct binding to the predicted sites (Fig. 5a), we generated reporters bearing single (mut187 or mut1342) and double (mut187 and mut1342, named Dmut) mutations in the predicted binding sites (Fig. S6). All mutant reporters abolished the miR-376a(e)-mediated repression (Fig. 5g). Thus, we concluded that miR-376a(e) directly binds the 3′ UTR of HLA-E at the predicted binding sites and that both binding sites are necessary for the regulation of HLA-E by miR-376a(e).

Finally, qRT-PCR analysis of the relative abundance of HLA-E mRNA in cells transduced with miR-376a(e) demonstrated no effect as compared to control cells (Fig. 5h), suggesting that miR-376a(e) represses HLA-E expression through translational inhibition.

### MiR-376a(e) regulation of HLA-E during HCMV infection

Because we demonstrated that ADAR1-p110 and editing of miR-376a are induced specifically following HCMV infection and since we showed that miR-376a(e) regulates HLA-E, we next tested whether miR-376a(e) controls HLA-E during HCMV infection.

**Figure 6:**
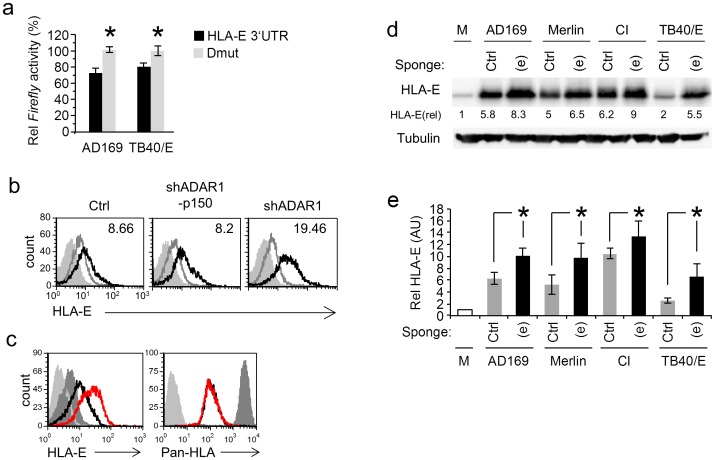
MiR-376a(e) regulates HLA-E expression during HCMV infection. (a) HFF and ARPE-19 cells were transfected with the indicated reporter plasmids and were infected with AD169 or TB40/E, respectively. Firefly/Renilla activity ratio was measured 48 hrs after infection. Data are average mean ± S.D. of three independent experiments; *P<0.05, by Student's t-test. (b) FACS analysis of HLA-E levels 96 hr after HCMV infection of HFF cells transduced with the indicated shRNA (black histogram). Empty grey histogram represents uninfected HFF cell, filled grey histogram represents background staining. The MFI of the black histogram is indicated on the top right corner. Data are a representative of three independent experiments. (c) FACS analysis of HLA-E levels and HLA class-I 96 hr after HCMV infection of HFF cells transduced either with control sponge (black histogram) and a specific anti-miR-376a(e) sponge (red histogram). Dark grey histogram represents uninfected HFF cell, light grey histogram represents background staining. Data are a representative of three independent experiments. (d) WB analysis of the levels of HLA-E 96 hrs following infection by various strains of HCMV (indicated above) of HFFs transduced either with a specific anti-miR-376a(e) sponge (labeled (e)) or with a control sponge (labeled Ctrl). Data are a representative of three independent experiments. Numbers indicate fold induction as compared to HLA-E expression in mock cells. (e) Quantification of all experiments performed in (d), shown are relative average intensities ± S.D., *P<0.05 by Student's t-test.

We initially validated that the miR-376a(e) binding sites in the 3′ UTR of HLA-E are targeted during HCMV infection. HFF and ARPE-19 cells were transfected either with the WT HLA-E 3′ UTR Firefly reporter or with the Dmut reporter and then the cells were infected with the AD169 (HFF cells) or the TB40 strains (ARPE-19 cells). The reporter's activity was repressed by both HCMV strains only when it was fused to the WT 3′ UTR of HLA-E and not when fused to the mutant 3′UTR (Fig. 6a).

We next proceeded to investigate whether ADAR1 has a role in the regulation of HLA-E during HCMV infection. To that end HFF cells were transduced either with shADAR1, with shADAR1-p150 or with a control, infected with HCMV and then the levels of HLA-E were assessed by FACS (Fig. 6b, quantified in Fig. S7a). HLA-E levels increased in all cells following infection (probably due to the binding of the UL40 leader peptide), however the shADAR1 transduced HFF cells showed an even higher expression of HLA-E (Fig. 6b, quantified in Fig. S7a).

We next wanted to demonstrate that miR-376a(e) specifically controls the endogenous expression of HLA-E during HCMV infection. For this we generated an anti-miRNA sponge construct, consisting of six adjacent binding sites for miR-376a(e), fused downstream to an eGFP cassette. Sponge constructs sequester the specific miRNA from its original targets, enabling their expression [40]. HFF cells were transduced with a control sponge (See Text. S1: Supporting Methods) or with an anti-miR-376a(e) sponge, and then infected with HCMV. The levels of HLA-E and of classical HLA class I proteins were analyzed by FACS (Fig. 6c, quantified in Fig. S7b). The expression of HLA-E was induced following infection however in HCMV infected HFF cells transduced with the anti-miR-376a(e) sponge the expression of HLA-E was significantly higher than that in the control cells (Fig. 6c, red histogram, quantified in Fig. S7b). Infections of all cells was similar as an equivalent reduction in the levels of classical HLA class I molecules was observed (Fig. 6c, right). Importantly, increased levels of HLA-E following transduction of HFF cells with the anti-miR-376a(e) sponge, were observed following infection with AD169, Merlin, CI and the TB40/E strain (Fig. 6d, quantified in 6e). Thus we concluded that following HCMV infection ADAR-p110 is induced, miR-376a is edited and that the edited miRNA, miR-376a(e), regulates the expression of HLA-E.

### MiR-376a(e)-mediated regulation of HLA-E during infection affects NK cell cytotoxicity

After we demonstrated that antagonizing ADAR1-p110 and miR-376a(e) affects HLA-E during HCMV infection we next wanted to test the functional implications of these findings.

HCMV infected HFF cells were initially transduced with the shADAR1 lentiviruses indeed showed reduced NK killing (data not shown). Yet, when the interaction between HLA-E and CD94 (the chain that recognizes HLA-E) were blocked, only a partial restoration of killing was observed (data not shown), suggesting that the ADAR1 proteins affect other molecules and pathways, in addition to HLA-E, that control NK cell activity.

**Figure 7:**
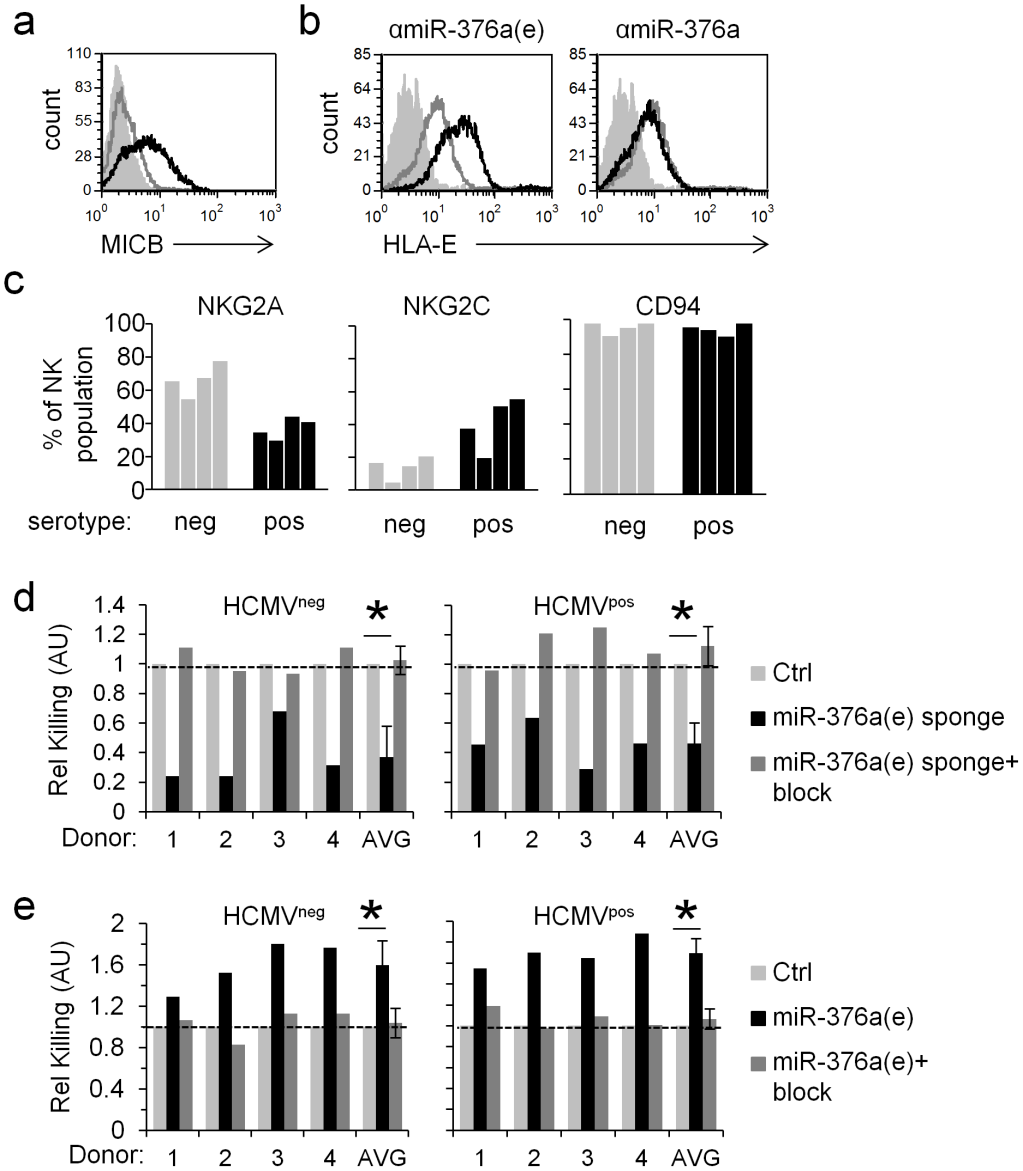
The regulation of HLA-E by miR-376a(e) during infection affects NK cell cytotoxicity. (a) FACS analysis of MICB levels on HFF cells transduced either with an anti-miR-376a sponge (black histogram), or an anti-miR-376a(e) sponge (grey histogram). Filled empty histogram represents background staining. Data are a representative of four independent experiments. (b) FACS analysis of HLA-E levels on HFF cells transduced either with a control sponge (empty grey histogram), an anti-miR-376a(e) sponge (black histogram, left), or an anti-miR-376a sponge (black histogram, right). Filled empty histogram represents background staining. Data are a representative of five independent experiments. (c) The percentage of NKG2A^+^ or NKG2C^+^ or CD94^+^ NK cells obtained from HCMV^neg^ (grey bars) and HCMV^pos^ (black bars) donors as detected by FACS. NK cells were identified by gating on CD56^+^CD3^−^ cells. (d and e) Killing of HCMV infected HFF cells transduced either with a control sponge or with a specific anti-miR-376a(e)-sponge (d) or with a control miRNA or miR-376a(e) (e). Killing was assessed 96 hrs post infection and was performed with bulk NK cells derived from four HCMV^neg^ and four HCMV^pos^ donors. The NK cells derived from the various donors were preincubated either with a CD94 blocking mAb (+block, dark grey bars), or with an isotype control (black bars) and then incubated with the ^35^S methaionine-labeled target cells. Data shown are the relative killing by each specific donor, and the relative average killing by all HCMV^neg^ or HCMV^pos^ donors combined (x-axis). The killing of the control-sponge (Ctrl, d) or control-miRNA (Ctrl, e) expressing HFF cells was set as 1 (marked by a dashed line). Shown are relative average killing ± S.D. *P<0.05, by Student's t-test. Of note, all single experiments also showed statistically significant effects (*P*<0.05, by Students' t-test) based on relative mean±S.D. of quadruplets.

Thus to demonstrate the functional effect miR-376a(e) has on NK killing of HCMV infected HFF cells we utilize the sponges mentioned above and for that we initially verified the sponge's specificity. HFF cells were transduced with the sponges against miR-376a and against miR-376a(e) and MICB levels were evaluated. In agreement with our above results in which we showed that miR-376a(e) does not control the expression of MICB (Fig. 4b), only the anti-miR-376a sponge had an effect on the levels of MICB in uninfected HFF cells (Fig. 7a). In contrast, during HCMV infection only the anti-miR-376a(e) sponge led to increase levels of HLA-E (Fig. 7b). We have also tested whether the sponges affect MICB expression following HCMV infection and observed that none of the sponges affect MICB expression during infection (Fig. S8). This is because the anti-miR-376a(e) sponge does not antagonize MICB (Fig. 7a) and because, as we have previously shown the anti-miR-376a sponge by itself is not sufficient to alter the levels of MICB during HCMV infection due to a synergistic interaction with the viral miR-UL112 [19].

HLA-E is the known ligand of two immune receptors; the inhibitory heterodimer CD94/NKG2A and the activating heterodimer CD94/NKG2C [15], [41]–[43]. It was demonstrated that the proportion of NKG2C^+^ NK cells increases significantly after HCMV infection [16], yet the functional significance of this expansion is still unclear. Thus, to test the functional significance of the miR-376a(e)-mediated control of HLA-E during infection we isolated NK cells from four HCMV^neg^ and four HCMV^pos^ donors (Fig. 7c). Consistent with previous reports [16], the proportion of the NKG2C^+^ NK population was indeed higher in the HCMV^pos^ as compared to HCMV^neg^ donors, while the proportion of NKG2A^+^ NK cells was lower in the HCMV^pos^ donors (Fig. 7c). In contrast, the proportion of CD94^+^ NK cells (CD94 is the common chain of the NKG2A and NKG2C heterodimers that recognizes HLA-E) did not significantly change between donors (Fig. 7c).

Next, HFF cells transduced either with the anti-miR-376a(e)-sponge or with a control sponge, were infected with HCMV and subjected to killing by NK cells derived from HCMV^neg^ and HCMV^pos^ donors. Irrespective of the donors' NKG2C^+^ NK cells percentage, the expression of the anti-miR-376a(e)-sponge which led to increased levels of HLA-E (Fig. 6c), resulted in inhibition of NK function and reduced killing of infected cells (Fig. 7d). Blocking of CD94 led to equivalent levels of killing of all cells by all donors (Fig. 7d). We also performed the reciprocal experiments in which we transduced HFF cells with miR-376a(e) (which leads to the down regulation of HLA-E). In this scenario, the killing of the infected HFF was increased (due to reduced inhibition) and as above, the increased killing was observed by all donors, irrespective of their phenotype (Fig. 7e). Blocking of CD94 restored the killing to that observed with cells infected by control miRNA (Fig. 7e).

Together, these results demonstrate the dominance of the inhibitory signal generated by NKG2A in controlling NK cell activity and the importance of the regulation of HLA-E by miR-376a(e) during HCMV infection.

### HCMV infection induces ADAR1 and reduces HLA-E expression in human decidua

We next wanted to investigate the influence of ADAR1-p110 induction on HLA-E expression in vivo. However, HCMV is very different from the mouse CMV (MCMV) and as the human virus infects only humans there is practically no murine model that enables us to investigate HCMV infection in vivo. Moreover, no sites for the mouse orthologue of miR-376a(e) (mmu-miR-376b(e)) were predicted in the 3′UTR of the mouse orthologue of HLA-E, Qa-1b.

HCMV is highly prevalent in the human population and is the leading cause of congenital infection, associated with severe birth defects and intrauterine growth retardation. The decidua is the maternal part of the placenta through which infection of HCMV is probably transmitted to the fetus. Recently, a new organ culture model was established in which human decidua is maintained, ex-vivo, and infected with HCMV [44]. This unique model enables us to examine some of our observations under physiological conditions.

**Figure 8:**
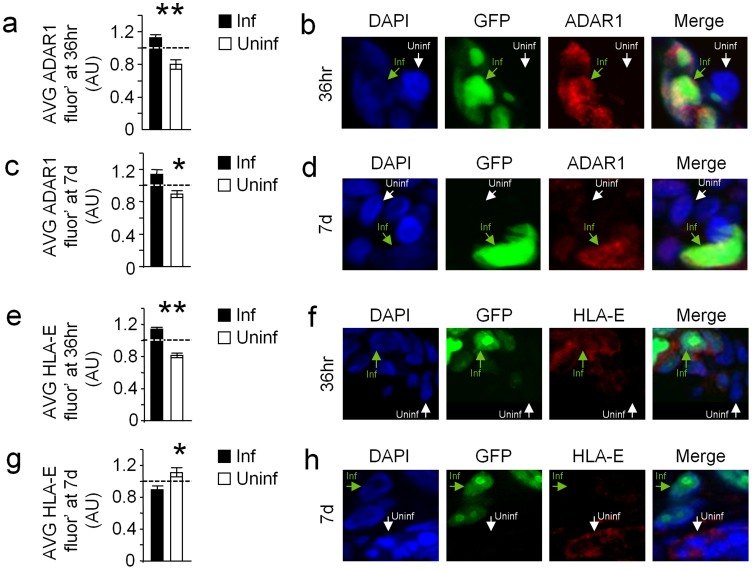
HCMV infection of human decidua organ culture induces ADAR1 and reduces HLA-E expression. (a–d) ADAR1 levels were assessed in decidua organ culture 36 hr (a and b) and 7days (c and d) after HCMV infection (Blue-DAPI, green-HCMV-infected cells, red-ADAR1). (a and c) Quantification of ADAR1 fluorescence intensity 36 hr (a) and 7 d (c) after infection. (a) Relative average ADAR1 intensity±SEM of GFP^neg^ cells (uninfected cells): 0.80±0.05, n = 123, Relative average ADAR1 intensity±SEM of GFP^pos^ cells (HCMV infected cells): 1.13±0.03, n = 123, *P<0.05, by student t-test. (c) Relative average ADAR1 intensity±SEM of GFP^neg^ cells (uninfected cells): 0.81±0.03, n = 143, Relative average ADAR1 intensity±SEM of GFP^pos^ cells (HCMV infected cells): 1.15±0.05, n = 109, *P<0.05, by student t-test. Data are summation of two independent experiments. (b and d) Representative examples. (e–f) HLA-E levels were assessed in decidua organ culture 36 hr (e and f) and 7days (g and h) after HCMV infection (Blue-DAPI, green-HCMV-infected cells, red-HLA-E). (e and g) Quantification of HLA-E fluorescence intensity 36 hr (e) and 7 d (g) after infection. (e) Relative average HLA-E intensity±SEM of GFP^neg^ cells (uninfected cells): 0.82±0.03, n = 193, Relative average HLA-E intensity±SEM of GFP^pos^ cells (HCMV infected cells): 1.14±0.03, n = 241, *P<0.05, by student t-test. (c) Relative average HLA-E intensity±SEM of GFP^neg^ cells (uninfected cells): 1.12±0.06, n = 70, Relative average HLA-E intensity±SEM of GFP^pos^ cells (HCMV infected cells): 0.85±0.04, n = 75, *P<0.05, by student t-test. Data are summation of two independent experiments. (f and h) Representative example.

Decidual organs were infected with the AD169 strain of HCMV that expresses IE72-GFP enabling the detection of infected cells (GFP^pos^). As expected, the HCMV infected cells (GFP^pos^) demonstrated typical enlarged cell morphology (Fig. 8). The organ cultures were next immunofluorescencenly stained for ADAR1 or HLA-E expression either early (36 hrs) or late (7days) during HCMV infection. In agreement with our above results, HCMV infected cells (GFP^pos^ cells) demonstrated elevated nuclear ADAR1-p110 staining at both early and late time points (Fig. 8a–d). In contrast, HLA-E levels were initially increased in HCMV infected cells (GFP^pos^, Fig. 8e and 8f), probably because of the viral UL40 protein, but were then reduced as infection prolonged (Fig. 8g and 8h), probably due to the editing of miR-376a(e) which results in the down regulation of HLA-E.

## Discussion

ADAR1 encodes two isoforms: ADAR1-p110, which was until now thought to be constitutively expressed, and ADAR1-p150, which is IFN-induced. These two isoforms are located at different compartments in the cell; ADAR1-p110 is located primarily in the nucleus, and ADAR1-p150 is located mainly in the cytoplasm [24]. It is therefore expected that each isoform will have a specific range of RNA targets, and thus different roles in different physiological conditions.

The role of ADAR1 proteins (especially ADAR1-p150) during infection was mostly investigated in the context of RNA viruses such as influenza virus [34], hepatitis C virus [35] Rift Valley fever virus [36] and mumps virus [37]. ADAR enzymes can also edit transcripts of DNA viruses (including viral miRNAs [33]), but in DNA virus infections it is unknown which ADAR protein mediates these editing events and what are the functional consequences of such editing.

Herein we demonstrate that the expression of ADAR1-p110 is induced during HCMV infection. We further show that this induction in ADAR1-p110 expression is HCMV specific and that it was not observed when other viruses were used. Additionally we show that miR-376a is edited during HCMV infection and that knockdown or over expression of ADAR1-p110 affects miR-376a editing, suggesting that ADAR1-p110 controls the editing of miR-376a during HCMV infection.

Whether ADAR1-p110 is induced by a viral component or by cellular is still unknown. We have shown that the kinetics of ADAR1-p110 induction is very rapid, and that the anti-viral drug Ganciclovir can not block this induction. In an attempt to investigated whether one of the immediate early genes of HCMV are responsible for the ADAR1 induction we expressed the viral transcription activators IE72 and IE86 in HFF cells, but did not observe any change in the levels of ADAR1-p110 (data not shown). Thus, further work is required to reveal whether it is a cellular or a viral component that is required for the ADAR1-p110 induction.

We also show that HLA-E, which was not known previously to be regulated by miRNAs, is downregulated by miR-376a(e) and that this consequently leads to the elimination of the HCMV infected cells by NK cells.

The question of why HCMV “allows” the induction of ADAR1 is interesting; particularly in view of the data we provided which show that the ADAR1-p110 induction is HCMV specific. It is possible that HCMV itself is dependent on cellular components of RNA editing for successful gene expression and infection (as in the case of viral miRNAs [45]) and thus can not evade the anti-viral response mediated by ADAR1-p110. Indeed it was shown that ADAR enzymes can edit transcripts of DNA viruses [33]. It is also possible that the virus developed mechanisms, which are yet unknown, to temper with the ADAR1-p110 response. This might be in similarity to what has been recently shown regarding IFI16 sensing of HCMV and the counter mechanism of the virus mediated by pUL83 [46]. Perhaps this balanced response enables the co-existence of the virus with its host.

As we have demonstrated that an UV-inactivated virus does not induce ADAR1-p110, it is tempting to speculate that a viral component controls ADAR1-p110 induction. Yet, it is also possible that upon live virus infection cellular-downstream elements trigger the induction of ADAR1-p110. Thus whether the induction of ADAR1-p110 is an anti-viral response or a viral-mediated tactic that the host turns to its advantage is yet to be determined.

Previously we showed that upon HCMV infection the virus downregulates the activating ligand MICB by the viral miRNA miR-UL112 [47]. We also demonstrated that miR-UL112 acts synergistically with the cellular miR-376a (that also regulates MICB), and that this leads to reduce killing of the infected cells [19]. Here we show that editing of miR-376a renders the infected cells susceptible to NK cell cytotoxicity. We were able to distinguish between these two seemingly contradicting observations because we used two different viruses. In all current experiments we had to use the AD169 laboratory strain of HCMV as similarly to clinical strains it encodes a UL40 protein with a leader peptide capable of being presented in the groove of HLA-E (GenBank accession number FJ527563). In our previous work we used the TB40/E strain that has a mutation in UL40 (GenBank accession number EF999921) and is unable to be presented by HLA-E and thus is unable to induce HLA-E expression [48]. Therefore, when TB40/E is used editing of miR-376a has no effect on HLA-E levels and we could nicely observe that the infected cells were less susceptible due to the viral and cellular miRNAs operating to reduce NK cell killing [19].

It is difficult to determine which strategy is superior to which, the viral down regulation of MICB or that of the host through increased editing and down regulation of HLA-E. During infection, both the virus and the host employ many mechanisms that affect the progress of infection. As HCMV is known to infect *in vivo* a wide rage of cells and furthermore as the virus has two modes of infection (latent and lytic) the question of who has the upper hand in this battle - the virus or the host, becomes very complicated. Thus, whether an infected cell will be killed or not depends not only on the specific cell in question but also on the mode and stage of infection as well as on the balance between the anti-viral and the viral strategies as shown here and in our previous publications [19], [49].

It was demonstrated here and previously that HCMV^pos^ individuals have increased proportion of NKG2C^+^ NK cells [16]. HCMV infection is a prerequisite for the expansion of these NKG2C^+^ NK cells as infection with other viruses, such as Hantaviruses, induces the expansion of the NKG2C^+^ population only in HCMV^pos^ individuals [49]. Although the expansion of the NKG2C^+^ NK cells is well established the role and functional significance of the NKG2C^+^ NK cells is still unclear and actually it is thought that it is only few NKG2C^+^ NK clones that expand [49]. Importantly, it was demonstrated that inhibition by NKG2A is dominant over the NKG2C activation [47]. Our current results are in line with these observations as we show that although the proportion of NKG2C^+^ cells is higher in HCMV^pos^ individuals, the outcome of neutralizing miR-376a(e) during infection (which leads to increased HLA-E levels) is inhibition of NK cell function irrespective of the donors' serotype.

The new mechanism of HLA-E regulation via miRNA editing discovered here is unique to humans. The mouse orthologous protein of HLA-E, Qa-1b, does not contain predicted binding sites for the mouse orthologue of miR-376a(e). Thus, to demonstrate the induction of ADAR1 and reduction in HLA-E expression following HCMV infection in physiological settings, we used a decidua organ culture model [44] and observed that during HCMV infection ADAR1-p110 expression is induced and that HLA-E expression is reduced. This unique system could be used in the future to study exciting questions regarding the relationships between the virus and its human host.

## Materials and methods

### Flow cytometry

The following antibodies were used: anti-hMICA (159227), anti-hMICB (236511), anti-hNKG2A(131411), anti-hNKG2C(134591) and anti-hCD94 (131412, blocking) all were purchased from R&D systems. The W6/32 hybridoma was purchased from ATCC. For HLA-E detection, the MEM-E07/08 antibodies were used (generously provided by V.H.),

### Generation of lentivirus, knockdown, overexpression and sponge vectors

Cloning and generation of lentiviruses and lentiviral vectors (ADAR1 knockdown, over-expression and sponge) was as previously described [50]. The specific sequences are listed in Text S1:Supporting Methods. The Drosha-knockdown vector and control vector were purchased from Sigma Aldrich and encode for puromycin resistance (HeLa cells were grown in the presence of 3.5 ug/ml puromycin).

### Viruses

In the present study, the following viruses were used: HCMV (strains: AD169, Merlin TB40/E and a clinical isolate), HSV1, HSV2, Adenoviruse, hMPV and Influenza (the A/PR8 strain).

HCMV UV inactivation prior to viral adsorption performed with the UV Stratalinker 2400 (StrataGene) at 0.99 Joule. Viral inactivation was confirmed in plaque assays.

### RNA extraction and cDNA preparation

Total RNA was extracted with TRI reagent (Sigma), and was treated with *Turbo*-DNase (Ambion). For generation of cDNA libraries the M-MLV reverse-transcriptase (Invitrogen) was used for reverse transcription (according to manufactures instructions), in the presence of a poly dT primer.

### Luciferase reporter assay

The 3′ UTRs of MICB and of HLA-E were cloned downstream to a *Firefly* reporter in the pGL3 vector (Promega) via the XbaI restriction site. Twenty-four hours prior to transfection of the reporter plasmids, cells were plated to 50% confluence in 24well plates, in triplicates. 200 ng/well of the pGL3 vector and 50 ng/well of the pRL-CMV vector were transfected with the TransIT-LT1 transfection reagent (Mirus Bio), and relative Firefly activity was assessed 48 hrs post transfection. Firefly/Renilla activity ratio was normalized to that in the control cells, and then relative activity of the reporter was calculated. The various ADAR1 promoter genomic regions were cloned upstream to a *Firefly* reporter via the XhoI and NheI restriction (promoters 1B, 1C and 2), or NheI and HindIII (promoter 1A) in the pGL4.14 vector (Promega). Forty-eight hours prior to transfection of the reporter plasmids, HFF cells were plated to 60% confluence in 24well plates, in triplicates. 250 ng/well of the pGL4.14 vector and 50 ng/well of the pRL-CMV vector were transfected with the TransIT-LT1 transfection reagent (Mirus Bio). Four hours after transfection, the media was removed, cells were washed, and media containing the reported treatment was added (mock, 1000 units/ml of IFN-α (Peprotech), 1000 units/ml IFN-β (Peprotech) or infection with AD169 strain of HCMV at MOI 1). Unless stated otherwise, Firefly activity was assessed 48 hrs post transfection. Firefly/Renilla activity ratio was calculated relatively to that in the mock cells. Primers for the amplification and mutations in the 3′ UTR of HLA-E and for the cloning of the various genomic regions are listed in Text. S1:Supporting Methods.

### Western blotting and quantification

Lysates of cells were prepared by lysing cells in ice-cold 0.6%SDS in 10 mM Tris (pH 7.5) buffer containing a cocktail of protease inhibitors (Roche). Lysate were separated using SDS–polyacrylamide gel electrophoresis and transferred to a NitroCellulose membrane. The following antibodies were used for protein detection: anti-hHLA-E (MEM-E02, generously provided by V.H.), anti-hADAR1 (SigmaAldrich, prestige antibodies, HPA003890), anti-hADAR2 (SigmaAldrich, clone ADAR2-8), anti-Drosha (Abcam, polyclonal), anti-αTubulin (Santa Cruz Biotechnology, B-7). Quantification of blots was performed with the ImageJ software.

### NK cell cytotoxcity assays

NK cells were isolated from healthy donors via MACS separation kit (Miltenyibiotech) and grown in the presence of IL-2 (peprotech). Target cells were grown over night in the presence of ^35^S added to a Methionine-free media (Sigma). Prior to incubation with the effectors, cells were washed, counted, and 5000 cells/well were plated. For each target, the spontaneous ^35^S release was calculated by cells which were not incubated with effector cells, and maximum ^35^S release was calculated by applying 0.1M of NaOH to the target cells. The level of ^35^S release was measured after 5 hours of incubation with effectors (at 37°_c_) by a β-counter TopCount (Packard). When blocking antibody was used, NK cells were preincubated with 0.5 ug/well of the blocking antibody on ice for 1 hour, and then the ^35^S labeled target cells were added for 5-hour incubation.

### Human decidual organ culture

Decidual tissues from women undergoing first-trimester elective pregnancy terminations were obtained by deep scraping to obtain maternal tissue from the basal plate and placental bed encompassing the decidua with interstitial trophoblastic invasion. The study was approved by the Hadassah Medical Center Institutional Review Board and was performed according to the Declaration of Helsinki, good clinical practice guidelines, and the human experimentation guidelines of the Israeli Ministry of Health. All donors gave written informed consent. Preparation of organ culture was as described [45]. For infection of decidual organ cultures, immediately after the sectioning the tissues were placed in 48-well plates (∼5 slices/well to maintain optimal viability) and were inoculated with the virus (40 PFU/well).

### Immunofluorescence, image processing and quantification

The following antibodies were used: anti-hHLA-E (MEM-E02, diluted 1∶500, generously provided by V.H.), anti-hADAR1 (SigmaAldrich, prestige antibodies, HPA003890, diluted 1∶650), detected by Goat anti-mouse DyLight647 or Goat anti-Rabbit DyLight647 (Jackson). Images were obtained using a Leica SP50 confocal microscope, using a 40× (1.25 NA) Leica oil objective. ImageJ software was used for image processing and quantification. Single cell intensities were normalized to the average fluorescence intensity of all cells after reduction of background intensity.

### Next generation small RNA sequencing data analysis

The Illumina HiSeq 2000 platform was used. All sequenced samples underwent initial pre-processing prior to differential expression analysis. Briefly, sequencing adapters were clipped and bases with quality lower than 30 were removed from both ends of each sequenced read. Reads shorter than 16 nucleotides after clipping and trimming were discarded. Remaining reads were mapped against the mature human miRNA reference sequences (downloaded from miRBase [51], allowing up to 2 mismatches between read and reference using BWA [52]. Reviewing the reads mapping to miR-376a (MIMAT0000729), a pileup of the supported alleles and their sequencing quality was produced using SAMtools [52]. The expected sequencing error rate was calculated per-position and a binomial cumulative distribution was implemented to differ significant modifications from sequencing errors.

## Supporting Information

Figure S1 **Analysis of ADAR genes expression during viral infection.** (a) WB analysis of ADAR2 expression in HFF and in 293T cells that served as a positive control. Alpha-tubulin served as a loading control. (b) WB analysis of the induction of ADAR1-p150 following IFN-α treatment (1000 u/ml). Numbers indicated relative induction of ADAR1-p150 expression compared to cells at time 0 hrs. Data are representative of three independent experiments. (c) Quantification of the experiments in (b). Shown are relative average intensities ± S.D. relative to cells at time 0 hrs. *P<0.001 by Student's t-test, ns - not significant. (d) Quantifications of all experiments performed in Fig. 1a. Shown is relative average intensity± S.D. The levels at time 0 hr were set as 1. *P<0.02 by Student's t-test. (e) Quantifications of all experiments performed in Fig. 1b. Shown is relative average intensity± S.D. The level of ADAR1-p110 in uninfected HFF cells was set as 1. *P<0.01 by Student's t-test. (f) Quantifications of all experiments performed in Fig. 1c. Shown is relative average intensity± S.D. The level of ADAR1-p110 in uninfected ARPE-19 cells was set as 1. *P<0.05 by Student's t-test. (g) Quantifications of all experiments performed in Fig. 1d. Shown is relative average intensity± S.D.. Levels in uninfected HFF cells were set as 1. (h) Quantifications of all experiments performed in Fig. 1e. Shown is relative average intensity± S.D. Levels in uninfected A549 cells were set as 1. *P<0.007 by Student's t-test. (i) Quantifications of all experiments performed in Fig. 1f. Shown is relative average intensity± S.D. Levels in uninfected A549 cells were set as 1. *P<0.002 by Student's t-test.
(TIF)

Figure S2 **KD efficiency by shADAR1-p150.** WB analysis of the KD efficiency of ADAR1-p110 and ADAR1-p150 by Ctrl, shADAR1 and shADAR1-p150, in HCMV infected cells. Numbers indicated relative expression of ADAR1-p110 compared to control KD (Ctrl). Data are representative of two independent experiments.
(TIF)

Figure S3 **Expression of NKG2D ligands and HLA-E on RKO cells.** (a) RKO cells were transduced with a control miRNA (empty grey histogram) or miR-376a (empty black histogram) and the levels of NKG2D ligands were assessed by FACS. Filled grey histogram represents background staining. (b) FACS analysis of the expression of HLA-E by RKO cells. HLA-E showed no expression on RKO cells.
(TIF)

Figure S4 **ADAR1 expression in cell lines and its effect on NKG2D ligands.** (a) The expression of ADAR-p110 and ADAR1-p150 in various cell lines were assessed by WB. ADAR1-p110 and ADR1-p150 were readily detected in the cell line examined. (b) BJAB and 293T cells were transduced with lentiviral vectors expressing either ADAR1-p110 (black histogram) or ADAR1-p150 (empty grey histogram) and the levels of the NKG2D ligands were assessed by FACS.
(TIF)

Figure S5 **Knock-down of Drosha.** (a) WB analysis of Drosha levels in HeLa cells transduced either with a control or with an shDrosha vector. Alpha-tubulin served as a loading control, numbers indicated the fold reduction compared to Drosha levels in cells transduced with a control vector. Data are representative of two independent experiments (b) Quantification of all experiments shown in (a). Shown are relative average intensities ± S.D. *P<0.01 by Student's t-test.
(TIF)

Figure S6 **Mutations in the 3′UTR of HLA-E.** Alignment of miR-376a(e) to the mutated 3′ UTRs of HLA-E which were fused downstream to the Firefly luciferase reporter. The mutated nucleotides are marked in red.
(TIF)

Figure S7 **Quantification of HLA-E levels during HCMV infection.** (a) Quantification of HLA-E expression in all experiments performed in Fig. 6b. Shown are relative average MFI ± S.D relative to Ctrl infected cells that were set as 1. *P<0.001, by Student's t-test. (b) Quantification of HLA-E expression in all experiments performed in Fig. 6c. Shown are relative average MFI ± S.D relative to mock cells that were set as 1. *P<0.005, by Student's t-test.
(TIF)

Figure S8 **Antagonizing miR-376a or miR-376a(e) does alters MICB expression during HCMV infection.** HFF cells transduced with an anti-miR-376a sponge (red histogram), anti-miR-376a sponge (back histogram) or a control sponge (filled dark grey histogram) were infected with HCMV and MICB levels were assessed by FACS 48 hrs after infection.
(TIF)

Text S1 **Supporting Materials and Methods.**
(DOC)
