# Supplementary figures and images for: MicroRNA Editing Facilitates Immune Elimination of HCMV Infected Cells
Source: PLoS Pathog. 2014 Feb 27;10(2):e1003963. doi: 10.1371/journal.ppat.1003963 (PMC3937316; doi:10.1371/journal.ppat.1003963)

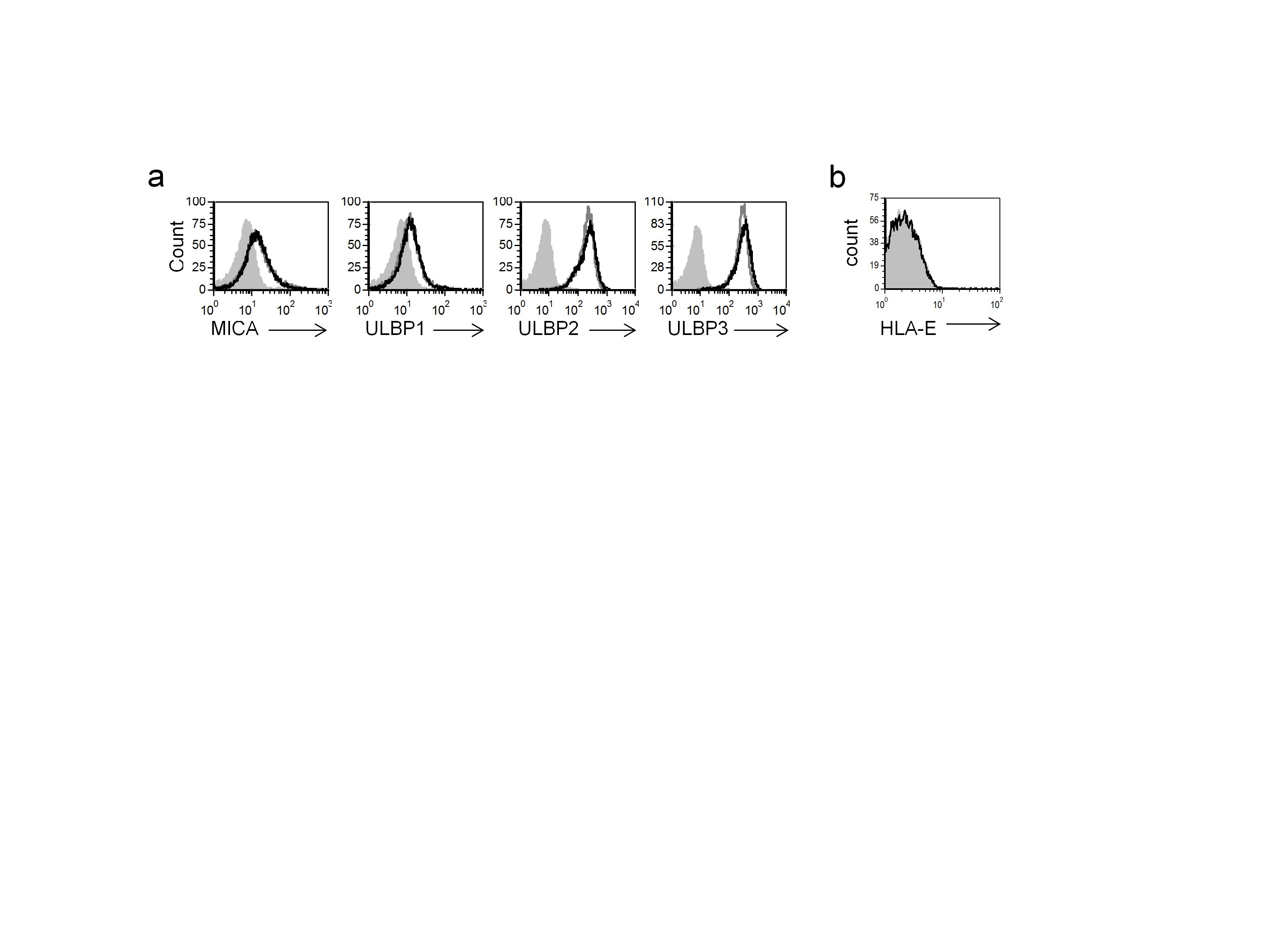

Supplement: Figure S3 — Expression of NKG2D ligands and HLA-E on RKO cells. (a) RKO cells were transduced with a control miRNA (empty grey histogram) or miR-376a (empty black histogram) and the levels of NKG2D ligands were assessed by FACS. Filled grey histogram represents background staining. (b) FACS analysis of the expression of HLA-E by RKO cells. HLA-E showed no expression on RKO cells. (TIF) [file ppat.1003963.s003.tif]

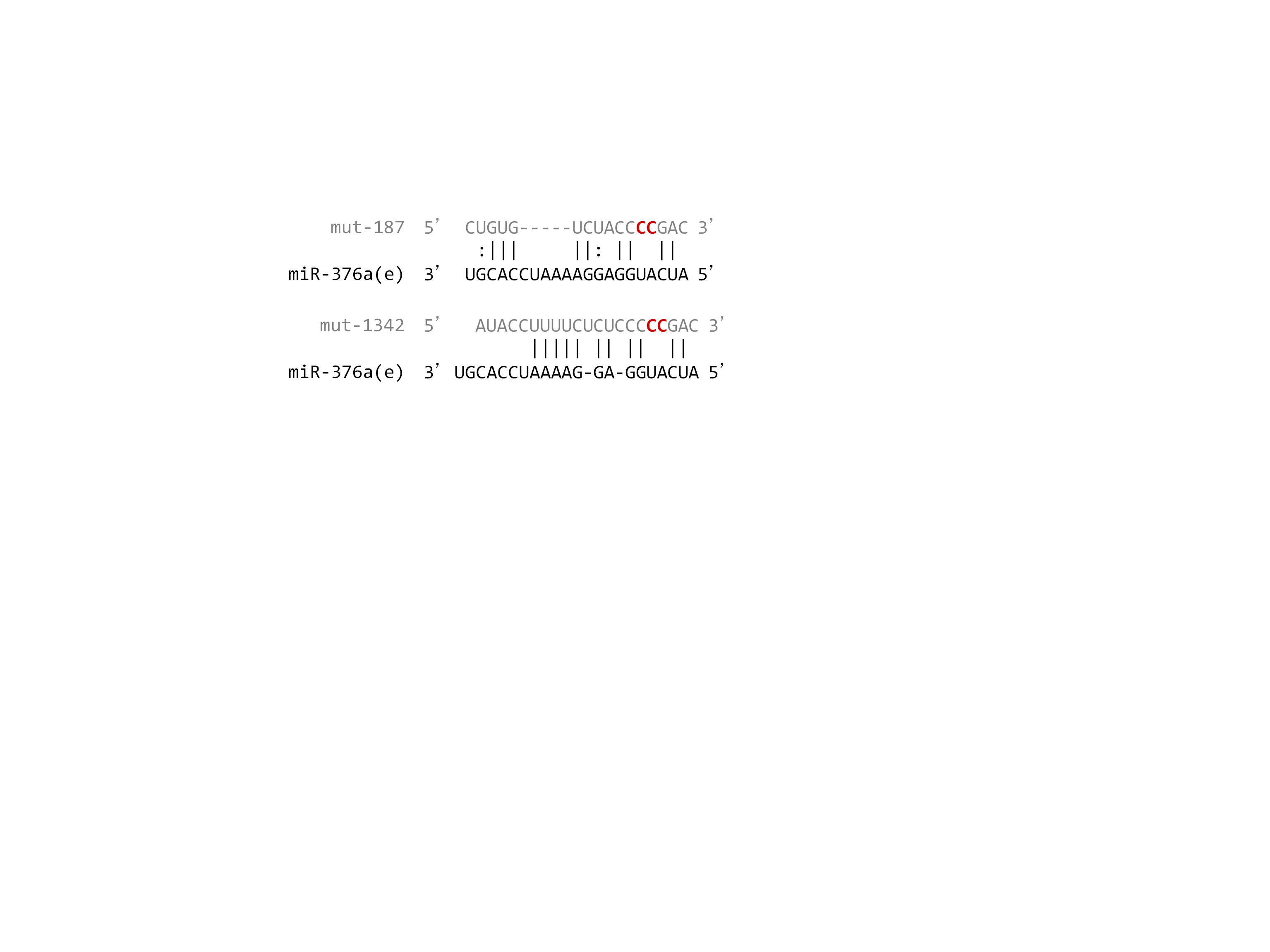

Supplement: Figure S6 — Mutations in the 3′UTR of HLA-E. Alignment of miR-376a(e) to the mutated 3′ UTRs of HLA-E which were fused downstream to the Firefly luciferase reporter. The mutated nucleotides are marked in red. (TIF) [file ppat.1003963.s006.tif]

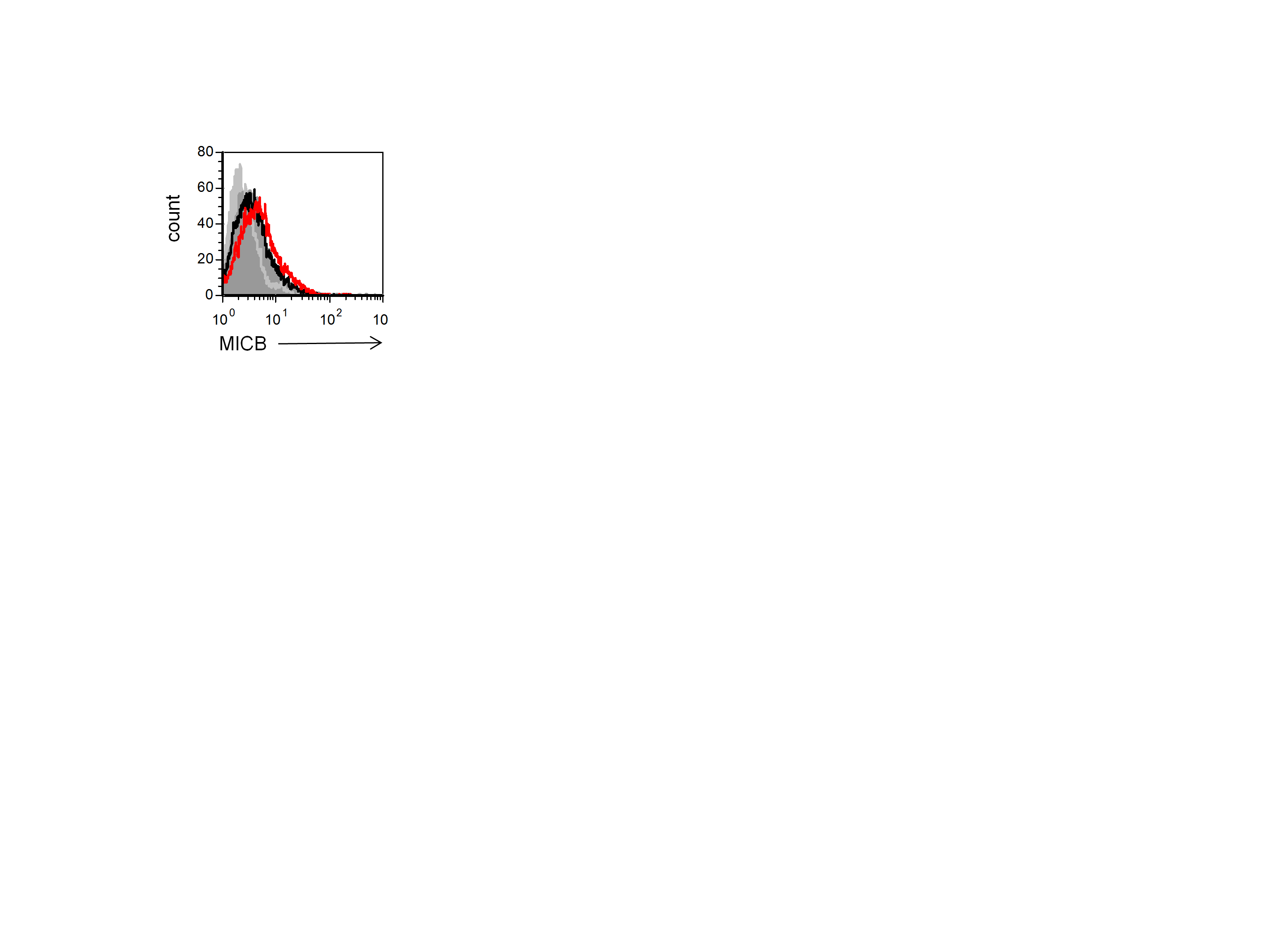

Supplement: Figure S8 — Antagonizing miR-376a or miR-376a(e) does alters MICB expression during HCMV infection. HFF cells transduced with an anti-miR-376a sponge (red histogram), anti-miR-376a sponge (back histogram) or a control sponge (filled dark grey histogram) were infected with HCMV and MICB levels were assessed by FACS 48 hrs after infection. (TIF) [file ppat.1003963.s008.tif]
