# Supplementary material for: MicroRNA Editing Facilitates Immune Elimination of HCMV Infected Cells
Source: PLoS Pathog. 2014 Feb 27;10(2):e1003963. doi: 10.1371/journal.ppat.1003963 (PMC3937316; doi:10.1371/journal.ppat.1003963)
Supplement: Text S1 — Supporting Materials and Methods. (DOC) [file ppat.1003963.s009.doc]

**Text S1. Supporting Material and Methods**

**Primers used in the current study**

| **shADAR1** | | | |
| --- | --- | --- | --- |
| Fw | GATCCCCCCAGCACAGCGGAGUGGUAttcaagagaUACCACUCCGCUGUGCUGGTTTTTGGAAA | | |
| Rev | AGCTTTTCCAAAAACCAGCACAGCGGAGUGGUAtctcttgaaUACCACUCCGCUGUGCUGGGGG. | | |
| **shADAR1-p150** | | | |
| Fw | GATCCCCGACCCGCGGAGUUUCCCGUttcaagagaACGGGAAACUCCGCGGGUCTTTTTGGAAA | | |
| Rev | AGCTTTTCCAAAAAGACCCGCGGAGUUUCCCGUtctcttgaaACGGGAAACUCCGCGGGUCGGG | | |
| **miRNA overexpression and Sponge constructs** | | | |
| Overexpression - miR-376a(e)-Fw | | GATCCCCATCATGGAGGAAAATCCACGTTTCAAGAGAACGTGGATTTTCCTCCATGATTTTTTGGAAA | |
| Overexpression - miR-376a(e)-Rev | | AGCTTTTCCAAAAAATCATGGAGGAAAATCCACGTTCTCTTGAAACGTGGATTTTCCTCCATGATGGG | |
| Anti-miR-376a(e) sponge binding sites | | ACGTGGATTAGGTCCATGAT | |
| Control miRNA-Fw | | GATCCCCCUAGGCGCGACUGAGAGAGCATTCAAGAGA TGCTCTCTCAGTCGCGCCTAGTTTTTGGAAA | |
| Control miRNA-Rev | | AGCTTTTCCAAAAACUAGGCGCGACUGAGAGAGCACGTTCTCTTGAATGCTCTCTCAGTCGCGCCTAGGGG | |
| Control sponge binding site | | GCGCCAGCCCCTGGGATCA | |
| **qPCR primers** | | | |
| GAPDH-Fw | | | AACAGCGACACCCACTCCTCC |
| GAPDH-Rev | | | CATACCAGGAAATGAGCTTGACA |
| HLA-E-Fw | | | TTGCTGCTGTGATATGGAGG |
| HLA-E-Rev | | | AGCTGTGAGACTCAGACCCC |
|  | | |  |
|  | | |  |
|  | | |  |
| HLA-E 3' UTR (into pGL3) | | | |
| Fw | | | AGCCTGAGACAGCTGCCTTG |
| Rev | | | CAAATGATGCTC AAACACAGCTT |
| Mut1 | | | CTCTGTGTCTACCCCGACCCC |
| Mut2 | | | TTTCTCTCCCCCGACCCTTTAAC |
| ADAR1 promoters genomic region (into pGL4.14) | | | |
| exon 1A- Fw | | | AATTTGACTTTTGTCCCTCTAGA |
| Exon 1A- Rev | | | CTGCCGCGG ATTCATTGCG |
| Exon 1B- Fw | | | TCTAGATGAGAGTTTGGAAATAC |
| Exon 1B- Rev | | | TCTAGAATTTAATGAAGTGTACAGC |
| Exon 1C- Fw | | | GTAAGCCGGGCCGGCCTT |
| Exon 1C- Rev | | | GGTTATTTTTTTTCTTGTGAGACG |
| Exon 2- Fw | | | GTGTGAGTACTATTCCATAGAATG |
| Exon 2- Rev | | | CGAGGTTCATGGGGTGGTCC |

**ADAR1 Overexpression**

| ADAR1-Fw | ATGGCCGAGATCAAGGAGAAAA |
| --- | --- |
| ADAR1-Rev | CTATACTGGGCAGAGATAAAAGT |
